# Clinical application of calcium silicate-based bioceramics in endodontics

**DOI:** 10.1186/s12967-023-04550-4

**Published:** 2023-11-25

**Authors:** Xinyuan Wang, Yizhi Xiao, Wencheng Song, Lanxiang Ye, Chen Yang, Yuzhen Xing, Zhenglin Yuan

**Affiliations:** 1 Department of Stomatology, Union Hospital, Tongji Medical College, Huazhong University of Science and Technology, Wuhan, 430022 China; 2 School of Stomatology, Tongji Medical College, Huazhong University of Science and Technology, Wuhan, 430030 China; 3 Hubei Province Key Laboratory of Oral and Maxillofacial Development and Regeneration, Wuhan, 430022 China

**Keywords:** Bioactivity, Bioceramics, Biocompatibility, Calcium silicate, Endodontics

## Abstract

Pulp treatment is extremely common in endodontics, with the main purpose of eliminating clinical symptoms and preserving tooth physiological function. However, the effect of dental pulp treatment is closely related to the methods and materials used in the process of treatment. Plenty of studies about calcium silicate-based bioceramics which are widely applied in various endodontic operations have been reported because of their significant biocompatibility and bioactivity. Although most of these materials have superior physical and chemical properties, the differences between them can also have an impact on the success rate of different clinical practices. Therefore, this review is focused on the applications of several common calcium silicate-based bioceramics, including Mineral trioxide aggregate (MTA), Biodentine, Bioaggregate, iRoot BP Plus in usual endodontic treatment, such as dental pulp capping, root perforation repair, regenerative endodontic procedures (REPs), apexification, root-end filling and root canal treatment (RCT). Besides, the efficacy of these bioceramics mentioned above in human trials is also compared, which aims to provide clinical guidance for their clinical application in endodontics.

## Background

Dental treatment is so related to our life that almost everyone has been to the dental clinic. Although regular oral healthcare had been recommended by the World Health Organization to prevent oral conditions [1, 2], the global burden of caries, especially untreated caries, is still high [3]. Untreated dental caries will cause pulpitis and then leads to further periapical periodontitis, which is an ordinary disease development process. According to some surveys [4, 5], periapical periodontitis is a frequently-occurring disease in the world, endodontic treatment is the most effective way to promote wound healing, which is determined by the selection of therapeutic methods and the related materials. With the development of materials science, the effects of endodontic treatment rely upon the applied material in endodontics, so calcium silicate-based bioceramics were introduced into the application of endodontic treatment in dental clinic.

Due to their predominant biocompatibility, bioactivity and sealing ability, calcium silicate-based bioceramics have been widely used in endodontic treatment, including MTA, Biodentine, Bioaggregate and iRoot BP Plus [6].

The most widely used and oldest calcium silicate-based bioceramic in dentistry was MTA [7]. MTA has favorable sealability and excellent biocompatibility. Furthermore, the continuous exposure in oral cavity could enhances the final setting of MTA. In addition, MTA can stimulate the generation of hydroxyapatite by releasing calcium ions to react with phosphorus [8, 9]. Therefore, MTA is commonly used for dental pulp capping, perforation repair, REPs, apexification, root-end filing and RCT [8]. However, the long setting time, high solubility at the early stage, tooth pigmentation and low compressive strength could be inconvenient to operate and lead to the failure of endodontic treatment [10–13]. Subsequently, auxiliary agents like calcium sulfate, calcium chloride, and propylene glycol were added to alleviate some of the shortcomings of MTA [14]. Furthermore, other novel kinds of calcium silicate-based bioceramics, including Biodentine, Bioaggregate, and iRoot BP Plus, were fabricated and applicated in dentistry. The primary constituents of Biodentine were tri- and di-calcium silicate without calcium aluminate, bismuth oxide and calcium sulfate which were present in MTA [15]. Although tricalcium silicate also existed in MTA, Biodentine had denser particles and less porous structures [16, 17]. As a result, Biodentine exhibited superior mechanical properties and shorter setting time compared to MTA. Also, the major components of Biodentine blended with calcium carbonate, oxide fillers, and iron oxide to achieve an optimal color [13, 18]. However, Biodentine exhibits a lower degree of radiopacity in comparison to other endodontic materials [19]. Consequently, alternative approaches may need to be employed to enhance the visualization of Biodentine in clinical contexts. The composition of Bioaggregate was akin to that of MTA. However, in Bioaggregate, tantalum oxide replaced bismuth oxide, thereby improving its color stability substantially. Moreover, Bioaggregate was aluminum -free material exhibiting lower cytotoxicity than MTA, rendering it a safer choice for clinical applications [20]. Also, Bioaggregate displayed better biocompatibility, bioactivity, compressive strength and acid resistance compared to MTA [21]. iRoot BP Plus boasted a shorter setting time in comparison to MTA [22]. Additionally, it displayed superior cellular viability and adhesion properties, which effectively facilitated tissue regeneration and optimal sealing performance [23–25], which contributed to its effectiveness in endodontic treatment. iRoot BP Plus also showed stronger acid resistance than MTA [26, 27]. The detailed characteristics of these calcium silicate-based bioceramics mentioned above have been summarized in our previous review [9]. Therefore, as different intervention measures require varying material properties, it is crucial to select the appropriate material for endodontic treatment in order to achieve optimal treatment outcomes.

Numerous studies have described the characteristics of these calcium silicate-based bioceramics and evaluated their applications in endodontics. Also, the different effects, biocompatibility and bioactivity of these bioceramics in cells and animals have been introduced by our previous reviews [9, 28]. However, there are still notable deficits in the clinical application of these materials due to the insufficiency of systematic summaries. Therefore, the present review aimed to analysis the clinical application, advantages and disadvantages of these bioceramics in patients from six aspects, including dental pulp capping, root perforation repair, regenerative endodontic procedures (REPs), apexification, root-end filling and RCT. The review seeks to provide guidance to clinicians in selecting the most appropriate calcium silicate-based bioceramics and related procedures in order to optimize endodontic treatment outcomes.

### Dental pulp capping

**Fig. 1:**
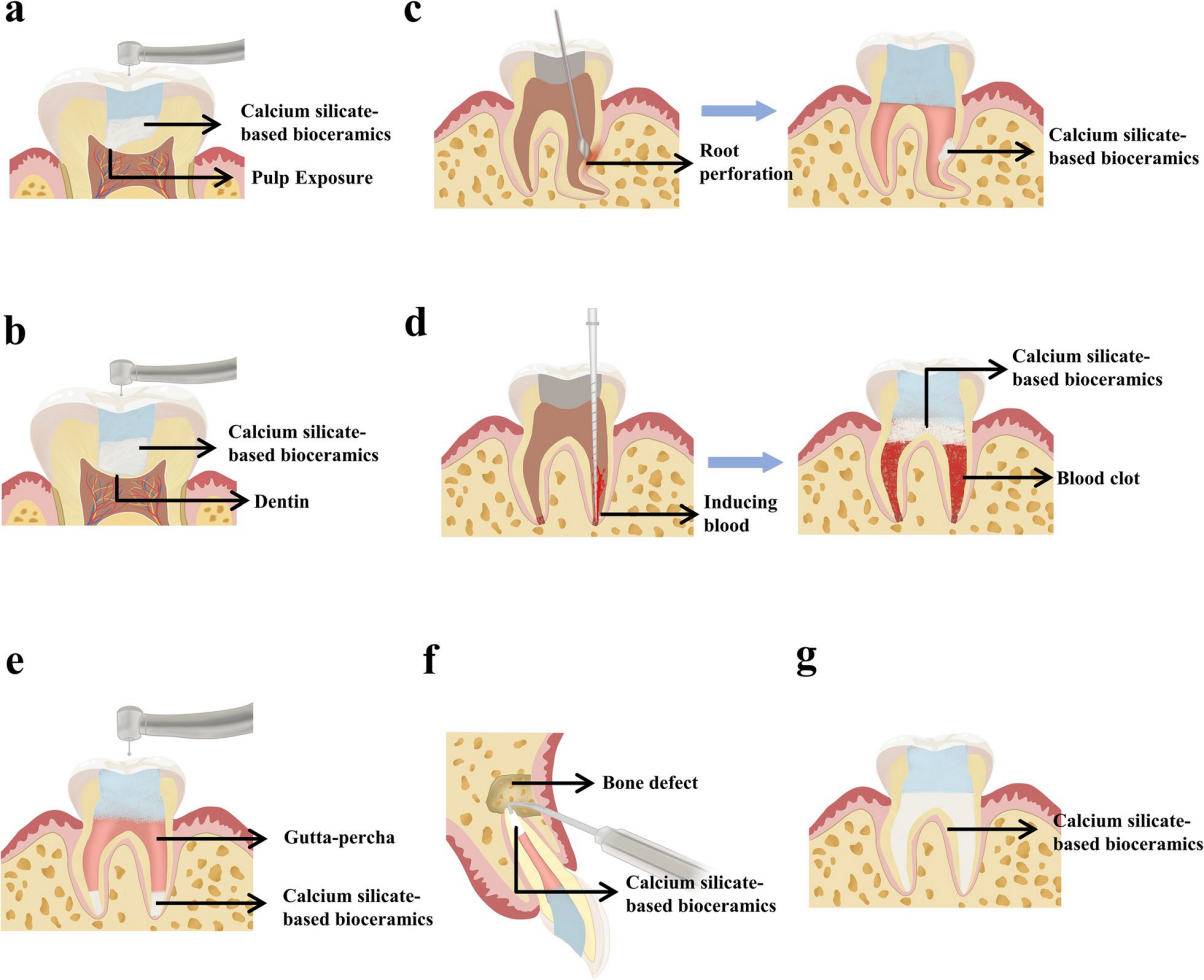
Schematic representations of endodontic treatment. **a** Direct dental pulp capping. **b** Indirect dental pulp capping. **c** Root perforation repair. **d** REPs. **e** Apexification. **f** Root-end filling. **g** RCT

Pulp capping is a method to protect vital pulp by covering pulp-near dentin or exposed pulp wound with bioactive materials to eliminate lesions, and it is divided into direct pulp capping and indirect pulp capping (Fig. 1a, b). Historically, calcium hydroxide (CH) was considered as the gold standard for pulp capping treatment, but CH was gradually replaced by calcium silicate-based bioceramics due to their excellent bioactivity, biocompatibility, sealing ability and mechanical properties [29]. MTA was one kind of calcium silicate-based bioceramic and possessed significant capacity to promote dentin formation of immature teeth and tissue regeneration [30], so it has been used earlier in the treatment of reversible pulpitis and pulp exposure. So far, a lot of researches have been performed to investigate the application of MTA in pulp capping.

It was proved that MTA could not only be used for direct pulp capping of pulp-exposed permanent teeth, but also for indirect pulp capping to reversible pulpitis [31]. Also, it was reported that dental pulp exposure resulting from caries and hypoplastic teeth with high healing potential was especially suitable for pulp capping with MTA [30]. Marques et al. [32] mentioned that the criteria for successful treatment with MTA were negative pulp cold temperature test, negative percussion, no subjective significant complaints, widened periodontal ligament and normal pulp vitality. Subsequently, Elzbieta et al. [33] demonstrated the superiority of MTA in indirect pulp capping treatment of deep carious lesions, and the results showed that MTA promoted faster pulp healing, thicker dentinal bridges, less inflammation, less hyperemia and necrosis, which attributed to the outstanding impermeability of MTA and the superior potential to prevent bacteria from infiltrating into pulp and triggering the inflammatory response [34]. Although a series of abnormal histological reactions, such as inflammatory infiltration, pulp necrosis, internal resorption, and irregular odontoblast cells, were observed, which might correlate with different effect sites of MTA and then result in the presence of regular and irregular odontoblast cells, MTA still displayed satisfactory healing ability from a clinical perspective, after direct pulp capping to infected deciduous teeth for five months [35]. Furthermore, pulp capping with MTA also displayed predictable high success rate in mature permanent teeth with less pain [36]. In addition, Cardoso-Silva et al. [37] reported the use of grey and white MTA in pulp capping of deciduous molars and indicated that both grey and white MTA possessed excellent biocompatibility, but more obvious dentin bridge could be observed in the presence of grey MTA during follow-up. Besides, to test whether the water-to-powder ratios of MTA could influence the effect of pulp capping, twenty-nine disease-free teeth were mechanically exposed, and filled with white MTA at different water-to-powder ratios of 0.28, 0.33 and 0.40, respectively. The result showed that there was no significant difference in the degree of dentine bridge formation and inflammatory response [38]. Moreover, to compare the effect of MTA with other pulp-capping materials in dental applications, a randomized clinical trial [39] was conducted in 50 patients aged 7−9 years and found that there was no significant difference in dentine bridge formation between MTA and Biodentine, which implied these two bioceramics possessed similar potential for maintaining pulp viability. Also, some systematic reviews have found high heterogeneity and generally do not observe differences among calcium silicate-based cements, such as MTA and Biodentine, in endodontic interventions. [40, 41]. Thus, MTA and Biodentine could be used not only as successful substitutes for CH but also as effective pulp capping materials. With the continuous development of materials, ProRoot MTA and MTA Angelus have emerged as two mainstream materials in the field of endodontic treatment. Of particular note is MTA Angelus, which has been demonstrated a shortened setting time of approximately 70% compared to the original MTA [42] and displayed similar histologic reaction in dental pulp capping [43]. Besides, some new materials were mixed with MTA to improve its properties. For instance, Lee, Li-Wan et al. [44] found that poly(ε-caprolactone) fiber mesh (PCL-FM) /MTA was more effective than MTA alone in pulp capping, which was evidenced by the formation of greater thickness of dentin bridges as well as no tooth discoloration. Also, some other novel calcium silicate-based bioceramics, such as Biodentine and Bioaggregate, were introduced into endodontic treatment.

Numerous studies reported that Biodentine displayed more excellent performance than MTA in endodontic treatment. Biodentine had the characteristics of shorter setting time (about 12 min) [45], easier handling and no teeth discoloration [29]. Hoseinifar et al. [46] applied Biodentine, MTA in direct pulp capping of human molars to compare the biological response of these materials. The results showed that the Biodentine exhibited higher rates of dentine bridge formation compared to MTA, which suggested that Biodentine might be an excellent alternative to MTA. However, the criteria for success of direct pulp capping were the capability of pulp capping material for restoring the vitality of the dental pulp rather than the degree of dentine bridge formation. Chompu-Inwai et al. [47] reported that there was no noticeable difference between Biodentine and ProRoot MTA in the application of pulping capping in cariogenic pulp exposure of permanent teeth. Moreover, a randomized controlled trial [39] showed that 100% success rate of direct pulp capping could be obtained with Biodentine or MTA, but the study possessed some drawbacks such as small sample size and short follow-up period. Interestingly, Mariusz Lipski et al. [48] found that the patients’ age might be correlated with tooth survival after pulp capping with Biodentine, and the age of 40 seemed to be a significant watershed of the prognosis of pulp capping. Some systematic reviews have found high heterogeneity and generally do not observe differences between MTA and Biodentine in dental pulp capping [40, 41]. Nevertheless, there is still a lack of high-quality clinical evidence to support this claim. Thus, more evidence for application of calcium silicate-based bioceramics in endodontic treatment is required to confirm theirs advantages. Bioaggregate also exhibits superior aesthetic outcomes with no grey discoloration and a shorter setting time [47].

As for iRoot BP Plus, some studies performed direct pulp capping with iRoot BP Plus to the mature permanent teeth with cariogenic pulp exposure. In 30 months, the prognosis of pulp capping was assessed on the basis of the teeth’s clinical symptoms and radiology examination. The results found that, although the success rate of pulp capping declined gradually during the three years, the overall success rate was still as high as 90% [49]. Besides, the effect of iRoot BP Plus as pulp capping material in pulpotomy was also investigated [50], and the results displayed that iRoot BP Plus could eliminate the pulp lesions with no tooth discoloration, but the success rate still seemed to significantly decrease over time. Therefore, more studies with long-term follow-up are required to further evaluate the effect of iRoot BP Plus in pulp capping.

Overall, MTA, Biodentine, Bioaggregate and iRoot BP Plus are all suitable materials for pulp capping (Table 1). They all process outstanding bioactivity and biocompatibility to promote the differentiation and proliferation of dental pulp cells and tertiary dentinogenesis [51–56]. Due to their high pH values, MTA, Biodentine, Bioaggregate, and iRoot BP Plus all exhibit good antibacterial properties [57–59]. Furthermore, they all have excellent sealability. MTA surface and dentin wall could achieve physical bond and chemical bond simultaneously to obtain undisputed sealability. In addition, Biodentine, Bioaggregate and iRoot BP Plus have better sealability than MTA [60–65]. Moreover, based on the absence of heavy metals found in MTA, Biodentine has demonstrated superior long-term efficacy in pulp capping compared to MTA [66]. Also, Biodentine is less susceptible to the influence of irrigating solutions than MTA [64]. Hence, in line with Kunert’s perspective [67], Biodentine is considered a more suitable material for pulp capping. There are few studies that comparing the specific advantages and disadvantages of other calcium silicate-based bioceramics in pulp capping.

**Table 1:** The major research findings of calcium silicate-based bioceramics in dental pulp capping

Application	Material	Main findings	References
Dental pulp capping	MTA	MTA could be used to replace CH because of its ability to promote tissue regeneration and dentin formation.	[29, 30]
		MTA possessed outstanding impermeability.	[33, 34]
		Grey MTA could induce more obvious dentin bridge than white MTA.	[37]
		There was no significant difference between MTA and Biodentine in dental pulp capping.	[39–41]
		MTA Angelus and ProRoot displayed similar biocompatibility, dentine bridge formation and histologic reaction, but MTA Angelus exhibited a shorter setting time.	[42, 43]
		Mixing PCL-FM with MTA could induce greater thickness of dentin bridges and no tooth discoloration.	[44]
	Biodentine	Biodentine possessed the characteristics of shorter setting time, easier handling and no teeth discoloration.	[29, 45]
		Biodentine could induce higher rates of dentine bridge formation compared to MTA.	[46]
		Both Biodentine and MTA could achieve similar success rate in pulp capping.	[39, 47]
		High heterogeneity and generally do not observe differences between MTA and Biodentine in dental pulp capping.	[40, 41]
	Bioaggregate	The aesthetic outcome and setting time of ProRoot MTA was not as excellent as Bioaggregate.	[47]
	iRoot BP Plus	The success rate of dental capping with iRoot BP Plus was 90% at 3 years follow-up and no tooth discoloration were observed.	[49, 50]

### Root perforation repair

During the process of root canal preparation and orthodontic mini-screws implant, one of the complications, root perforation, could result in the inflammation of periodontal tissue and calcium silicate-based bioceramics could be used to repair it (Fig. 1c). MTA was applied to repair the perforation successfully and the inflammation of periodontal tissue induced by root perforation was generally inhibited [68], which was similarly observed in two cases during a five-year follow-up [69]. In addition, a historical cohort study found that MTA appeared to be a long-term stable sealant for root perforation regardless of the location of MTA in the 12 to 107 months of follow-up [70]. Mente, Johannes et al. [71] reported that the outstanding performance of MTA could be attributed to the high concentration of calcium released from MTA. Regarding the long-term efficacy of MTA on the treatment of root perforation, a 10-year follow-up prospective cohort study [72] was performed in which 110 patients with single root perforation were selected, and the results showed that only 8% cases displayed little or no healing. The main cause of such failure included that, the patients above the age of 50 years, the perforation position located in the middle of root, and the perforation area larger than 3 mm, which might lead to the difficulty of inflammatory site to regain full healing. Furthermore, Pace et al. [73] repaired the furcal perforation with a matrix-free MTA, no discomfort was reported by the patients and the radiographic examination showed no periapical lesion and normal biological function of the tooth at the follow-up five years later. However, the success rate of root perforation repair with MTA was reduced among the patients with older root perforation and furcal perforation.

As mentioned previously, owing to the characteristics of MTA, such as low cytotoxicity, no mutagenic potential, and the induction of the formation of mineralized tooth tissue, MTA should be an ideal material in root perforation repair. Plenty of clinical trials described the treatment of inadvertent perforation or caries-related forked perforation with MTA. It was found that there was no significant difference between Pro-MTA and Angelus MTA when either of them was used to repair the furcal perforation, but white MTA displayed better esthetic effect than grey MTA [74]. Furthermore, MTA showed excellent performance in moist environments due to its hydrophilia, which could be proved by no negative effect on MTA in the presence of blood contamination [75]. Therefore, MTA was an ideal calcium silicate-based bioceramic to repair the furcal perforations.

There were few clinical literatures available about the application of Biodentine in root perforation repair. It was reported that Biodentine could be used to repair the multiple perforations of the first mandibular molar with an extensive resorptive lesion [76]. After 18 months, no uncomfortable clinical symptoms were observed. After 43 months, the function of the tooth was well preserved, and the root was not broken. However, it was found that the adhesion ability of Biodentine was weaker than that of MTA after blood contamination. For example, Üstün et al. [75] collected 96 mandibular molars with forked perforation to investigate whether blood contamination could affect the anti-malposition ability of root perforation repair materials. The results showed that the excellent adhesion ability of MTA still existed, but Biodentine displayed poor adhesion strength due to the blood contamination. Interestingly, when uncontaminated by blood, Biodentine exhibits superior bonding strength compared to MTA, attributed to its smaller particle size [64].

A randomized controlled trial [77] was performed to compare the effect of Bioaggregate and MTA in root perforations repair. The 2 years’ follow-up radiographic analysis showed both Bioaggregate and MTA displayed excellent clinical efficacy in the repair of the perforation of the molars pulp floor which caused by the accidental or iatrogenic trauma.

Only an in vitro study [65] was carried out to compare the performance of iRoot BP Plus and MTA in root perforation repair. The study collected 40 newly extracted molars to create the root perforation model and detected leakage value by measuring the glucose concentration in the leakage. The results showed that iRoot BP Plus group displayed lower microleakage and tighter material-periodontal ligament tissue junctions. Therefore, it seemed that iRoot BP Plus possessed more excellent characteristic than MTA when used in root perforation repair, which should be proved by further in vivo experiments.

In summary, in human trials of root perforation repair materials, MTA and Biodentine have been extensively studied (Table 2). MTA is one of the most widely used materials in root perforation repair due to its ability to release calcium ions that promote the repair of periodontal tissues and its long-term stability [70, 71]. For Biodentine, studies have shown that it has similar effects to MTA in no blood contamination situation, but its adhesive strength is lower than MTA’s in contaminated environments [64]. However, it was more negatively affected by blood contamination as compared to MTA [75]. iRoot BP Plus has been demonstrated lower microleakage and a tighter connection with periodontal ligament tissue [65], but further in vivo experiments are needed to confirm it. All in all, MTA is currently the best choice for root perforation repair, especially in complex situations such as blood contamination. While Biodentine and iRoot BP Plus also have good features, more experimental data is needed to support their application in the field of root perforation repair.

**Table 2:** The major research findings of calcium silicate-based bioceramics in root perforation repair

Application	Material	Main findings	References
Root perforation repair	MTA	MTA could repair the perforation and control the inflammation of periodontal tissue.	[68, 69]
		MTA possessed long-term stable sealant, biocompatibility and ability of the release of high concentration of calcium.	[70, 71]
		MTA displayed satisfactory long-term efficacy.	[72, 73]
		There was no significant difference between Pro-MTA and Angelus MTA in furcal perforation repair, but white MTA displayed better esthetic effect than grey MTA.	[74]
		ProRoot MTA and RetroMTA still existed more excellent adhesion ability than Biodentine and Supra MTA in blood contamination setting.	[75]
	Biodentine	Biodentine demonstrates superior bond strength when compared to MTA, but it is more susceptible to the influence of blood contamination.	[64, 75, 76]
	Bioaggregate	Bioaggregate and grey MTA displayed similar satisfactory clinical efficacy.	[77]
	iRoot BP Plus	iRoot BP Plus displayed lower microleakage and tighter material-periodontal ligament tissue junctions than MTA.	[65]

### Regenerative endodontic procedures (REPs)

Apexification is a traditional method to treat pulpitis and periapical periodontitis of immature permanent teeth, but there are still several drawbacks when apexification was performed in endodontic treatment, such as resting root development, thinner dentine wall, and increasing likelihood of root fracture. REPs are a promising and alternative method to treat pulpitis and apical periodontitis of immature permanent teeth. The general procedures of REPs include properly disinfecting the root canal first, then stimulating periapical tissue to induce blood from the periapical area and form a blood clot as a scaffold in the root canal space. Finally, biocompatible materials are placed onto the coronal part of the root canal to prevent it from coronal microleakage (Fig. 1d). In the past, the regeneration of pulp tissue of infected non-vital teeth was impossible, but REPs subvert this perception. The success of REPs could be evaluated by some evidences after treatment, which included an increase in root length, thickening of the canal walls, and detection of dental pulp activity. In 2008, a pilot clinical study performed by Shah et al. [78] discovered that the final outcomes of many cases met the evaluation criteria for successful REPs, which implied that REPs possessed the potential to promote the root development continually [79–81]. However, some other studies came to the opposite conclusion [82, 83], which might be caused by the failure of blood clot formation and obvious postoperative pain due to improper disinfection. In general, the success rate of REPs was relatively high and the prognosis seemed to be satisfactory.

MTA was widely used in REPs due to its excellent characteristics especially its favorable biocompatibility and sealing ability. In addition to the periapical inflammation caused by dental caries and pulpitis, the combined use of REPs with MTA was also effective in the treatment of complex root fractures and root resorption perforation [84–87]. Although REPs were primarily applied in the treatment of infected young permanent teeth, they could also be effective in the treatment of infected mature permanent teeth. For example, Saoud et al. [88] performed REPs on infected mature permanent teeth in patients aged 8–21 years, and REPs could eliminate not only uncomfortable clinical symptoms but also pulp and periapical inflammation. Moreover, Paryani et al. [89] introduced two case reports which displayed similar results and the patients in these two cases were 11 and 14 years old respectively. In addition, it was found that REPs could be used as an alternative after the failure of MTA apical plug in permanent teeth [90]. The satisfactory results might be attributed to active metabolism and uninfected vital pulp cells, epithelial root sheath of Hertwig, as well as periodontal ligament/apical papilla stem cells of younger patients. Remarkably, a mixture of MTA and blood in REPs could lose its radio-opacity over time [87]. Although the underlying mechanism was not clarified, it was speculated that some substances related to radio-opacity in MTA might be degraded after mixing MTA with blood. Furthermore, Jun et al. [91] investigated the therapeutic effects of the location of MTA on the upper part and the lower part of the root after REPs. The results suggested that there was no significant difference between the apical part and the coronal part of the root. However, root in the apical part developed faster than that in the coronal part in both the early and late stages, which was able to prevent the tooth discoloration caused by MTA, but it might limit the ingrowth of pulp-like tissues and the preservation of dental pulp vitality. Therefore, improved procedures of REPs with MTA might lead to optimal results and increase the success rate of REPs. The severest disadvantage of MTA was the tooth discoloration [92, 93], and this problem could be solved by increasing the times of appointment to remove MTA and then restoring the tooth surface with the composite resin [94].

Due to its excellent properties, such as satisfied consistency, lower cytotoxicity, less discoloration, shorter setting time, and no apical displacement during condensation [95], Biodentine was also applied in REPs and could promote the development of immature roots in endodontic treatment [96, 97]. A controlled clinical study was performed to compare the efficiency of white MTA and Biodentine in REPs, and the results showed that there was no significant difference between them in respect of root length, pain and sinus occurrence. However, less discoloration of the tooth could be observed in the presence of Biodentine [98], which suggested Biodentine might be a substitute for MTA in REPs. Surprisingly, Chaniotis et al. [99] reported a clinical case that REPs were performed to an immature fractured tooth with Biodentine, which was followed by orthodontic treatment, and there was no obvious defects to the tooth. To improve the efficiency of Biodentine in REPs, growth factors were also added together to enhance the effect of Biodentine on the success rate of REPs. For example, Bakhtiar et al. [100] found that the addition of PRF (platelet-rich fibrin) in the blood clot scaffold and sealing canals with Biodentine could promote further development of the root, which was similar to the efficiency of Biodentine with PRP (platelet-rich plasma) in REPs. [101] In addition, Cordero et al. [102] mixed allogeneic umbilical cord mesenchymal stem cells with platelet-poor plasma and then sealed the root canals with Biodentine, which could preserve non-vital immature teeth caused by apical periodontitis and root perforation. Similarly, Meza et al. [103] performed REPs in mature permanent teeth via the mixture of dental pulp stem cells and pulp-derived leukocyte platelet-rich fibrin (L-PRF), followed by sealing with Biodentine, the results displayed that all vitality tests of teeth were standard except cold response. All these studies mentioned above could shed more light on new ideas to treat infected mature teeth and teeth with difficult formation of blood clots.

The clinical application of Bioaggregate and iRoot BP Plus in REPs has not been extensively studied. However, Bioaggregate and iRoot BP Plus have been observed to promote odontoblastic differentiation and the formation of mineralization nodules in vitro [104, 105]. Therefore, further investigation is warranted to explore the potential of Bioaggregate and iRoot BP Plus in REPs, as they may also demonstrate promising results.

Based on current clinical research, as we mentioned before, due to the great biocompatibility, bioactivity, and sealing ability of Biodentine and MTA, both materials could be used for REPs [51, 95] (Table 3). However, Biodentine had better aesthetic results compared to MTA [13, 18], but its cost and patient acceptance still need to be considered. Additionally, they can be combined with growth factors to achieve better clinical outcomes [100, 101].

**Table 3:** The major research findings of calcium silicate-based bioceramics in REPs

Application	Material	Main findings	References
REPs	MTA	The root continual development could be found after REPs with MTA.	[79–81]
		REPs could eliminate uncomfortable clinical symptoms, pulp and periapical inflammation.	[88, 89]
		REPs with MTA was an alternative method to MTA apical plug.	[90]
		Mixing MTA with blood might decrease the detrimental effect of MTA on radiographic evaluation.	[87]
		The location of MTA on the apical part could induce faster root development, less tooth discoloration, but inhibition of the ingrowth of pulp-like tissues and the preservation of dental pulp vitality.	[91]
		Tooth discoloration caused by MTA could be solved by removing MTA and composite resin restoration.	[92–94]
	Biodentine	Biodentine excellent properties, including satisfied consistency, lower cytotoxicity, less discoloration, shorter setting time, and no apical displacement during condensation.	[95]
		Biodentine could be applied in REPs promote the development of immature roots.	[96, 97]
		There were no obvious different in the therapeutic efficacy between white MTA and Biodentine, but Biodentine could induce less tooth discoloration.	[98]
		The tooth after treatment by REPs with Biodentine could withstand the forces generated by the orthodontic devices.	[99]
		Mixing growth factors, such as PRF, PRP, platelet-poor plasma, L-PRF, with blood clot could improve the the efficiency of REPs with Biodentine.	[100–103]
	Bioaggregate and iRoot BP Plus	Bioaggregate and iRoot BP Plus have been observed to promote odontoblastic differentiation and the formation of mineralization nodules in vitro.	[104, 105]

### Apexification

If the dental pulp of immature teeth encountered irretrievable inflammation or necrosis, apexification could be used to induce the formation of root apex for the subsequent RCT (Fig. 1e). In brief, apexification induced the apical formation via the root canal preparation, disinfection, and filling with CH. Then patients need to make an appointment with the dentists every 3–6 months until that radiographic examination revealed the accomplishment of the root canal closure and calcified tissue deposition in root canal.

Giuliani et al. [106] firstly introduced MTA into the use of apexification, in which the mixture of MTA and amalgam was placed on the root apex after disinfection, and then the canals were filled with thermoplastic gutta-percha after one week. The results displayed that the formation of hard apical tissue could be observed after three appointments. However, it should be noted that the MTA in these cases was mixed with amalgam, and according to the authors, this combination could have a beneficial effect on root closure. Further research is needed to confirm the effectiveness and safety of this approach for apexification. Furthermore, when the acute periapical abscess was developed in the replanted immature permanent tooth, apexification with MTA was used for eliminating the periapical radiolucency [107, 108]. Due to its outstanding sealing ability, it was reported that the apexification with MTA reduced severe periapical lesions caused by dens invaginatus [109, 110]. Apexification with MTA in necrotic immature permanent teeth has been performed in plenty of cases [111–114]. Remarkably, Songtrakul et al. [115] proposed a modified apexification method: 3 mm thickness of absorbable collagen matrix could be delivered into the location of the root apex and then MTA/Biodentine was placed on it, and the upper part of the root canal was filled with gutta-percha, which led to an increase in root length, the thickness of root wall, root apex closure and the generation of pulp-like tissue. It was speculated that the mechanism involved in such result was that inflammation-induced granulation tissue formation and periapical tissue healing could induce the migration of stem cells to the location of root apex, which promoted the development and mature of the root apex. Similarly, PRF was applicated in the procedure of apexification before the placement of MTA, which could decrease the failure rate of apexification successfully [116]. Based on the studies mentioned above, absorbable materials and platelet-derived growth factors such as PRF could be applied with MTA in the procedure of apexification to improve the success rate of apexification. Furthermore, several case reports manifested that it was unnecessary to manage the displacement of MTA by surgery if the healing of periapical lesions could continue by itself, only regular follow-up was required [117, 118]. However, Demiriz et al. believed that the extrusion of MTA might delay the healing of the periapical lesion [119], which might be due to the fact that the extrusion of MTA was able to cause persistent chronic inflammatory stimulation and adverse effect on the periodontal tissue [120]. Root fracture was one of serious complications in apexification. Çiçek et al. [121] elucidated that the thickness of MTA could influence the resistance of tooth fracture and 3-millimeter (mm) thickness of MTA was considered the optimal thickness to obtain excellent fracture resistance. And apexification with MTA followed by post-core and crown could restore immature anterior teeth and achieve sufficient fracture resistance [122]. In addition, the prognosis of replanting tooth after apexification with MTA seemed satisfactory, but tooth discoloration and root resorption was easily observed after orthodontic treatment. The underlying mechanism how orthodontic treatment induced root resorption after apexification was poorly understood and required to be further explored [123].

MTA was commonly used in apexification, but its most prominent disadvantage was tooth staining [124]. Biodentine was found to exhibit similar effects to MTA in apexification, and its application in apexification was capable of obtaining satisfactory aesthetical outcome [125]. Furthermore, Biodentine could lead to the deposition of hydroxyapatite crystals on the interface between the material and the dentin, which helped to provide outstanding sealing ability [126]. Therefore, Biodentine could be an excellent substitute for MTA in apexification [127]. Besides, Sharma et al. [126] found that Biodentine could be mixed with PRF in apexification. It was worth noting that the subjects of one case was a teenager and the other two cases were adults aged 39 and 45, which implied that this approach was useful in both underage and adult patients. Moreover, Lertmalapong et al. [128] compared the effects of different materials’ thickness on bacterial leakage and marginal adaptation in apexification, and the results showed that either 3 mm thickness of Biodentine or 4 mm thickness of ProRoot MTA was the optimal thickness to obtain the ideal treatment outcome of apexification. However, Sogukpinar et al. [129] proved that, no matter what kind of material was used in apexification, teeth fracture resistance tended to decrease over time.

In conclusion, both MTA and Biodentine could be used in apexification (Table 4). MTA has excellent sealing ability, but its most prominent drawback is tooth discoloration [124]. Biodentine exhibits similar effects to MTA in apexification procedures, and provides better aesthetic outcomes, especially suitable for the treatment of anterior teeth [125]. Additionally, when mixed with PRF, Biodentine can achieve better treatment results [126]. Furthermore, the impact of material thickness on apexification treatment outcomes requires further investigation, and the optimal thickness of the material for apexification should be determined. In clinical practice, various factors should be weighed to select the most suitable material for the patient.

**Table 4:** The major research findings of calcium silicate-based bioceramics in apexification

Application	Material	Main findings	References
Apexification	MTA	Mixing MTA with amalgam might improve root closure.	[106]
		Apexification with MTA possessed excellent sealing ability and could reduce severe periapical lesions.	[107–110]
		The application of absorbable materials and platelet-derived growth factors before the placement of MTA could elevate the success rate of apexification.	[115, 116]
		The extrusion of MTA might delay the healing of periapical lesion, but it was unnecessary to remove MTA by surgery, if the healing of periapical lesions could continue by itself.	[117–120]
		3 mm thickness of MTA followed by post-core and crown repair could achieve better fracture resistance.	[121, 122]
	Biodentine	Biodentine displayed better aesthetical outcome and induced more deposition of hydroxyapatite crystals on the interface between the material and the dentin.	[125, 126]
		3 mm thickness of Biodentine or 4 mm thickness of ProRootMTA could achieve the lowest bacterial leakage and the best marginal adaptation in apexification.	[128]

### Root-end filling

Root-end filling is an effective method to manage the necrotic pulp and periapical lesions of teeth [130–132] (Fig. 1f). Due to its excellent sealing ability and prominent bioactivity in bone regeneration, MTA was also widely applied in root-end filling [133, 134]. Arx et al. [135] conducted a 10-year follow-up study to prove the efficacy of MTA as a root-end filling material, but to display decreased teeth fracture resistance relative to the healthy tooth.

With the development of dental materials, numerous retrospectives or randomized controlled studies were performed to compare the effect of MTA with other materials on root-end filling. For instance, compared to zinc-free amalgam, intermediate restorative material and Super-Ethoxybenzoic acid, MTA could resist bacteria microleakage for the longest period, which explained the feasibility of MTA as a root-end filling material [136, 137]. The most robust sealing ability of MTA had also been demonstrated through electrochemical testing by Mark et al. by Martell et al. [138]. In addition, Subramanyam et al. [139] found that blood and saliva pollution had no effect on the compressive strength of MTA, and MTA mixed with blood appeared to display higher compressive strength. Further, occlusal loading played a detrimental role in the edge adaptability of the material, Peters et al. [140] also reported that MTA could be applied in the complex human masticatory system because of outstanding marginal adaptation after loading. In terms of cure rate of MTA, it was proved by a 5-year follow-up clinical study that the cure rate of MTA could achieve 92.5% in root-end filling. [141]. Moreover, MTA was also found to exhibit satisfactory rapid healing dynamics [142]. Nevertheless, Aydın et al. [143] questioned the role of MTA in the root-end filling, due to the fact that MTA was not significantly related to the increased trabecular bone, which did not align with the advantages of MTA mentioned before. The contradiction might be caused by these studies’ differences in population, inclusion criteria, the ability of the operator, the size of the lesion, the tooth group and so on.

Besides the tooth discoloration, MTA possessed some other drawbacks as root-end filling material. For example, bismuth oxide was added to MTA for its radiopacity, which led to more porosity and less mechanically resistant over time [15, 144]. Thus, another kind of calcium silicate-based bioceramic without bismuth oxide, Biodentine, was created as root-end filling material to solve this problem [13]. As to the clinical application of Biodentine in root-end filling, Caron et al. [18] applied Biodentine as root-end filling material for endodontic surgery and the results showed that the periapical lesion healed after one year and bone regeneration was observed after two years. Also, in animal experiments, Biodentine had been shown to exhibit better sealing ability than MTA in root-end filling [145]. Nevertheless, Ramezanali et al. [146] found that there was no significant difference in microleakage among MTA, and Biodentine. In terms of iRoot BP Plus, Zhou et al. [147] performed a prospective randomized controlled study to compare the effect of iRoot BP Plus and MTA on the treatment outcome of endodontic microsurgery. The results displayed that iRoot BP Plus possessed comparable potential to enhance the healing of periapical tissue as root-end filling material.

Root-end filling materials should possess good biocompatibility and facilitate healing by interacting with stem cells in the periapical tissues [58]. All of the materials we mentioned possess those properties. In summary (Table 5), MTA, as a widely used root-end filling material, has achieved outstanding therapeutic effects in the treatment of periapical lesions, but there are some drawbacks such as discoloration and low mechanical resistance [124, 144]. Biodentine is a new type of calcium silicate-based bioceramics, which can effectively promote bone tissue regeneration [18]. Hence, Biodentine is one of the more superior root-end filling materials available. iRoot BP Plus is a newly developed root-end filling material that shows potential in promoting periapical tissue healing [147], although its efficacy needs further clinical verification. Although Bioaggregate has been demonstrated the promising bioactivity and biocompatibility, as well as possessing characteristics necessary for a root-end filling material [9, 28], there has been limited research on its application in both animal and clinical studies. Consequently, further investigations are required to confirm its efficacy in root-end filling.

**Table 5:** The major research findings of calcium silicate-based bioceramics in root-end filling

Application	Material	Main findings	References
Root-end filling	MTA	MTA could decrease teeth fracture resistance.	[135]
		The bacteria microleakage rate of MTA was lower than zinc-free amalgam, intermediate restorative material, Super-EBA and EndoSequence Bioceramic Root Repair Material.	[136, 137]
		The compressive strength of MTA would not be affected by blood and saliva pollution.	[139]
		MTA possessed better sealing ability than Super EBA, and IRM materials.	[138]
		Both MTA and EBA possessed outstanding marginal adaptation.	[140]
		Root-end filling with MTA displayed higher cure rate than that with adhesive resin composite, IRM, silver amalgam.	[141, 142]
		Root-end filling with MTA led to more porosity and less mechanically resistant than that with Portland cement.	[15, 144]
	Biodentine	Biodentine could eliminate periapical lesion and stimulate bone regeneration in root-end filling.	[18]
		Biodentine had been shown to exhibit better sealing ability than MTA in root-end filling.	[145]
		There was no significant difference in microleakage among MTA and Biodentine.	[146]
	iRoot BP Plus	iRoot BP Plus was comparable with MTA in root-end filling.	[147]

### Root canal treatment(RCT)

RCT is a versatile method to treat irreversible pulpitis and periapical periodontitis in endodontics [148] (Fig. 1g). Root canal treatment involves procedures of mechanical preparation and chemical flush to remove infection from the root canal followed by the root canal filling and sealing of the crown [149]. Although the method of gutta-percha filling could lead to a success rate of RCT between 83% and 97.1% [149], it may not be as effective in retreatment cases and in treating refractory periapical inflammation due to its complex canal anatomy and the difficulty in completely eliminating bacteria.

Calcium silicate-based bioceramics have been demonstrated to be suitable for root canal filling, due to its biocompatibility, bioactivity and antibacterial properties. As a root canal filling material, MTA exhibits superior sealing properties compared to gutta-percha [150].

For instance, Kim et al. [151] demonstrated the excellent sealing properties of MTA for root canal filling using a glucose penetration model in extracted human teeth. This characteristic of MTA was attributed to the formation of a hydroxyapatite layer at the interface between MTA and dentin, which was facilitated by the long tags formed by the MTA surface coming into contact with dentinal tubules. This study also further confirmed the biomineralization ability of MTA. George et al. [152] reviewed the use of MTA in root canal filling and their case studies showed significant healing, normal tooth function, and absence of symptoms after 4 years of follow-up, as confirmed by X-ray images. Furthermore, the study conducted by AlJasser et al. [153] demonstrated that the combination of MTA root canal filling and bone grafting procedures significantly improves endo-perio lesions. The authors attributed the phenomenon to the notable properties of MTA, particularly its capacity to facilitate and expedite cellular differentiation, proliferation, and induce tissue formation.

Besides, it is worth noting that after RCT, teeth often exhibit increased fragility. The increased resistance to root fracture had been demonstrated after root canal with MTA [154]. Likewise, Girish et al. [155] discovered that employing MTA for complete root canal filling could yield superior resistance to fracture when compared to the use of MTA solely for apexification followed by gutta-percha filling. Additionally, MTA exhibited strong antimicrobial properties, particularly against Enterococcus faecalis, which could be commonly isolated in refractory periapical periodontitis [58]. Moreover, MTA also could resist the leakage of Enterobacter aerogenes and Staphylococcus epidermidis [156].

In clinical practice, Ferreira et al. [157] conducted a study indicating that the use of MTA for root canal filling would not result in postoperative pain. Additionally, Terauchi et al. [158] discovered that, similar to gutta-percha, overfilling or flush root filling with MTA would lead to any detrimental effects. However, insufficient filling with MTA could increase the likelihood of nonhealing and necessitate surgical intervention. Therefore, the aforementioned studies have provided validation for the use of MTA as an alternative material to gutta-percha in root canal filling.

However, studies have demonstrated that MTA, when used as a root filling material, could exhibit some degree of porosity, which could be attributed to the difficult manipulation of MTA and the different technique employed during obturation [154, 156]. An et al. [154] proposed that utilizing a Ni-Ti file with a reverse motion technique for obturation could help mitigate porosity compared to the conventional manual compaction method. Additionally, Lawley et al. [156] reported that employing ultrasonic energy during MTA placement could achieve improved results.

In vitro experiments, Biodentine had exhibited an elastic modulus similar to dentin, which allowed it to reinforce fragile root structures. Furthermore, studies have shown that the compressive strength of Biodentine could increase over time gradually and ultimately approached the compressive strength of natural tooth dentin (297 MPa) [155]. Additionally, there is a research has reported that iRoot BP Plus possessed significant and comparable antibacterial activity against Enterococcus faecalis, similar to MTA [58].

Although these in vitro experiments provide preliminary information regarding the properties of Biodentine and iRoot BP Plus as root filling materials, a direct comparison of their clinical effects is absent due to limited clinical research. Further clinical studies are necessary to comprehensively evaluate the efficacy of novel calcium silicate-based bioceramics, such as Biodentine, Bioaggregate, and iRoot BP Plus, as root filling materials and determine their applicability in clinical practice.

In summary, calcium silicate-based bioceramics show great promise as root filling materials in RCT (Table 6). However, further research and technological advancements are needed to address the challenges associated with these materials, such as handling difficulties and potential porosity. Nonetheless, their unique properties, including excellent sealing ability, promotion of biomineralization and tissue formation, fracture resistance, and antimicrobial activity, make them attractive alternatives to traditional gutta-percha.

**Table 6:** The major research findings of calcium silicate-based bioceramics in RCT

Application	Material	Main findings	References
RCT	MTA	MTA exhibits superior sealing properties compared to gutta-percha.	[150, 151]
		After 4 years of follow-up, the apical periodontitis tooth treated with MTA root canal filling showed significant healing, normal function, and remained symptom-free.	[152]
		The combination treatment of root canal filling with MTA and bone grafting has been shown to effectively improve the condition of endo-perio lesions.	[153]
		Root canal filling with MTA has shown to increase resistance to root fracture compared to gutta-percha.	[154, 155]
		MTA possesses the ability to combat Enterococcus faecalis, Enterobacter aerogenes, and Staphylococcus epidermidis to reduce the probability of root canal retreatment.	[58] [156]
		No postoperative pain was observed after root canal filling with MTA.	[157]
		The quality requirements for root canal filling with MTA were similar to those for gutta-percha.	[158]
		The technique used for MTA root canal filling could influence the porosity rate.	[154, 156]
	Biodentine	Biodentine had a similar elastic modulus to dentin, reinforcing fragile root structures.	[155]
	iRoot BP Plus	iRoot BP Plus exhibited significant and comparable antibacterial activity against Enterococcus faecalis, similar to MTA.	[58]

## Conclusion and perspective

Due to its excellent biocompatibility, enhanced sealing ability, satisfied bioactivity calcium silicate-based bioceramics have been widely applied in endodontic treatment, such as dental pulp capping, root perforation repair, REPs, apexification, root-end filling and RCT. To improve the efficiency of calcium silicate-based bioceramics in endodontic treatment and minimize the possible side effects on the patients, plenty of clinical case reports were introduced to investigate the treatment outcome of these calcium silicate-based bioceramics in endodontic treatment. In addition, to compared the clinical efficiency of these calcium silicate-based bioceramics in endodontic treatment, lots of randomized controlled clinical studies have been carried out to examine their advantages and disadvantages in the application in endodontic treatment.

In this review, the clinical application of four main calcium silicate-based bioceramics, including MTA, Biodentine, Bioaggregate and iRoot BP Plus, in endodontic treatment was summarized. Firstly, the characteristics of calcium silicate-based bioceramics were emphasized in terms of their biocompatibility, sealing ability, bioactivity, and other related aspects. Furthermore, given that there has been some novel calcium silicate-based bioceramics that emerged in recent years, their clinical application in endodontic treatment was also proved by many studies. Nowadays, although there have been a large number of studies about the clinical application of calcium silicate-based bioceramics in endodontic treatment, there is still great scope for further investigations toward their properties. Moreover, except for MTA which is considered the gold stand among various calcium silicate-based bioceramics in endodontic treatment, relatively few studies were performed to test the clinical efficiency of other calcium silicate-based bioceramics in endodontic treatment. In addition, inconsistencies in results across different research groups may be attributed to factors such as sample size, patients’ gender and age, severity of pulp/periapical lesions, evaluation criteria, follow-up period, and other variables. Therefore, the establishment of standardized guidelines and evaluation criteria is necessary for future studies to obtain more scientific and reliable conclusions.

However, this review still has several limitations. Firstly, there is a lack of high-quality clinical evidence, especially large-scale randomized controlled clinical trials, resulting in a limited understanding of the efficacy and long-term prognosis of these materials. Secondly, their differences and molecular mechanisms are not discussed in detail. The reason could be attributed to that clinical literature often focuses on comparing the differential effects of materials in clinical practice, making it challenging to obtain human samples for evaluation of the underlying molecular mechanisms.

Future developments in calcium silicate-based bioceramics for endodontic treatment include material improvement, combination applications, and customized treatment. Research efforts could focus on updating the physical, chemical, and biological properties of the materials. Moreover, exploring combination applications with other materials could further augment their functionalities and effects. Tailoring the formulation of these materials based on individual patient conditions and requirements may enhance treatment precision and outcomes. Through continued research and innovation, calcium silicate-based bioceramics will better address clinical demands and assume a more prominent role in endodontic treatment.

## Data Availability

Not applicable.

## Abbreviations

MTA: Mineral trioxide aggregate
REPs: Regenerative endodontic procedures
RCT: Root Canal Treatment
CH: Calcium hydroxide
PCL-FM: Poly(ε-caprolactone) fiber mesh
PRF: Platelet-rich fibrin
PRP: Platelet-rich plasma
L-PRF: Leukocyte platelet-rich fibrin
mm: Millimeter
